# Smartphone based colorimetric method for determination of memantine via schiff’s base reaction with ascorbic acid

**DOI:** 10.1038/s41598-026-51216-4

**Published:** 2026-05-12

**Authors:** Samar H. Elagamy, Aya Barseem

**Affiliations:** 1 Department of Pharmaceutical Analytical Chemistry, Faculty of Pharmacy, Tanta University, Tanta, Egypt; 2 Pharmaceutical Analysis Department, Faculty of Pharmacy, Menoufia University, Menoufia, Egypt; 3 Department of Pharmaceutical Analytical Chemistry, Faculty of Pharmacy, Menoufia National University, Km Cairo-Alexandria Agriculture Road, Menofia, Egypt

**Keywords:** Memantine hydrochloride, Colorimetry, Spectrophotometry, Ascorbic acid, Smartphone, ImageJ, Pharmaceuticals, Biological techniques, Chemistry

## Abstract

The smartphone-based colorimetric approach has emerged as a powerful analytical tool with broad applications, enabling fast and cost-effective measurements, eco-friendly operation with low energy demand, and reasonable analytical performance. In this work, a simple and eco-friendly colorimetric method was developed for the determination of memantine (MEM) in pharmaceutical dosage forms. In this approach, MEM reacts with ascorbic acid to produce a pink-colored product exhibiting two absorption maxima (λ_max) at 386 nm and 530 nm. The color response was captured using a smartphone camera and analyzed digitally with ImageJ software, where the extracted color intensity values were correlated with MEM concentration. A conventional spectrophotometric method relying on the absorbance at 386 nm was also established. Both methods exhibited excellent linearity over the concentration range of 5.0–50.0 µg/mL. The smartphone-based method showed a limit of detection (LOD) of 0.45 µg/mL and limit of quantitation (LOQ) of 1.36 µg/mL, while the spectrophotometric method exhibited a LOD of 0.0615 µg/mL and LOQ of 0.19 µg/mL. The greenness of the methods was evaluated using the newly introduced EPPI tool, a comprehensive metric that assesses greenness, applicability, and analytical performance. The results are presented as pie charts and numerical scores. The total scores for the smartphone and colorimetric approaches were 84.4 and 82.4, respectively, indicating the superiority of the smartphone-based method in terms of sustainability and practical applicability.

**Supplementary Information:**

The online version contains supplementary material available at 10.1038/s41598-026-51216-4.

## Introduction

Memantine (MEM) is chemically known as 3,5-dimethyladamantan-1-amine) Figure S1. It is an N-methyl-D-aspartate (NMDA) receptor antagonist and was first approved by the FDA in 2013 for the management of Alzheimer’s disease. MEM is a white crystalline powder that is freely soluble in water, with a melting point of approximately 292–295 °C (hydrochloride salt). MEM possesses moderate lipophilicity (logP ≈ 3.3) and a low molecular weight (215.76 g/mol for the hydrochloride salt). The compound is a weakly basic drug with an estimated pKa of approximately 10.7.

Owing to the absence of a chromophore, MEM lacks native absorbance in the UV–visible region and does not exhibit intrinsic fluorescence. Therefore, several spectrophotometric and spectrofluorimetric methods have been developed for its determination based on derivatization or complex formation with chromogenic or fluorogenic agents. Spectrophotometric approaches include reactions with 1,2-naphtoquinone-4-sulfonic acid sodium salt (NQS)^1^, quinalizarin (Quinz), p-chloranilic acid (p-CA), and 7,7,8,8-tetracyanoquinodimethane (TCNQ)^2^, ninhydrin and ferric chloride^3^, N, N-Dimethyl Aniline^4^ as well as ion-pair complexation with dyes such as rose bengal^5^. Spectrofluorimetric methods rely on reactions with fluorogenic reagents such as acetyl acetone and formaldehyde^6^ or o-phthaldialdehyde (OPA)^7^, or by employing fluorescent nanomaterials like red-emitting BSA–copper nanoclusters^8^. Electrochemical analysis has also been reported using molecularly imprinted polymer (MIP)-based electrochemical sensors^9,10^. Moreover, HPLC methods have been utilized for the separation of MEM, with detection performed by UV^11,12^, fluorimetry after derivatization^13–15^, or mass spectrometry (MS)^16^. HPTLC methods have also been reported for its simultaneous determination with donepezil^17,18^.

The smartphone-based colorimetric approach has emerged as a highly versatile and powerful analytical tool with broad applications across diverse fields^19–26^. This approach generally involves two key steps: (1) image acquisition using the smartphone camera and (2) color quantification via specialized image-processing software (Adobe Photoshop, ImageJ, or MATLAB,) within a selected color space. The relationship between the extracted color data and analyte concentration is then established, and results can be visualized or interpreted using dedicated mobile applications. Several color spaces (models) are used in image analysis, including RGB, CMYK, XYZ, Lab*, HSV, and grayscale models. Among these, RGB, HSV, and grayscale are the most widely employed due to their simplicity and effectiveness in quantitative evaluation^27^. ImageJ is a Java-based, open-source software developed by the National Institute of Mental Health. It is one of the most commonly used platforms for such analyses. It supports multiple image formats and can quantify color intensity by measuring the RGB gray levels of colored substances^28^. The RGB gray value represents pixel intensity on a scale from 0 (black) to 255 (white). These RGB values can be mathematically converted into CMY (cyan, magenta, yellow) values using the equation CMY = 255 – RGB, where CMY values are directly proportional to color intensity^29,30^. Moreover, smartphone applications such as Colorimeter can display various color models (RGB, CMYK, HSV) and allow real-time extraction of color values from any point on the screen, enhancing the convenience and practicality of smartphone-based colorimetric analysis^31^.

The aim of this work is to develop a simple and reliable colorimetric method for the quantitative determination of MEM in pharmaceutical dosage forms. The proposed approach involves the application of smartphone-based colorimetry using ImageJ software to analyze the colorimetric response generated from the reaction of MEM with ascorbic acid. The novelty of this work lies in presenting the first smartphone-based colorimetric analysis of MEM. Unlike previously reported derivatization-based approaches, the proposed method is greener and more sustainable, requiring minimal energy and employing ascorbic acid as an eco-friendly reagent. Experimentally, the method features a simple and straightforward procedure, with well-defined reaction conditions in DMF that ensure complete derivatization. The derivatized product is stable, allowing reliable colorimetric measurement, and the digital analysis via ImageJ provides accurate quantification of color intensity. Besides its simplicity and eco-friendly nature, the method achieves analytical sensitivity comparable to conventional spectrophotometric approaches, making it suitable for routine pharmaceutical analysis.

## Experimental

### Apparatus

Spectrophotometric analysis was performed using a Shimadzu UV-1800 double-beam spectrophotometer (Shimadzu, Japan) equipped with 1.0 cm quartz cuvettes. Measurements were recorded within the visible spectral range at a data interval of 0.1 nm.

### Materials and reagents

Memantine Hydrochloride MEM and Ascorbic acid ASC were purchased from Sigma-Aldrich (USA) Dimethylformamide (DMF, 99%) was procured from Alpha Chemika (Egypt). Alzmenda^®^ tablets (Labeled to contain 10 mg MEM) were obtained from a local pharmacy.

### Preparation of stock and working solutions of memantine

A stock solution of MEM was prepared by dissolving 25 mg of the drug in 25 mL of distilled water. From this stock, a working solution of 100 µg mL⁻¹ was prepared using DMF as the diluting solvent.

### Preparation of standard solution of ascorbic acid

ASC solution (0.2% w/v) was obtained by dissolving 100 mg of ASC in 0.5 mL of distilled water, and the final volume was made up to 50 mL with DMF.

### Derivatization procedure for memantine

Aliquots ranging from 0.5 to 5.0 mL of MEM standard solution in DMF (100 µg mL⁻¹) were accurately transferred into screw-capped glass tubes. To each tube, 2.0 mL of the ASC solution (0.2%) was added. The tubes were then heated in a water bath at 100 ± 5 °C for 30 min, followed by cooling to room temperature. The resulting reaction mixtures were quantitatively transferred into 10 mL volumetric flasks and diluted to the mark with DMF to yield final concentrations in the range of 5.0–50 µg mL⁻¹. All reaction tubes were tightly sealed during heating to prevent solvent evaporation, and no significant volume loss was observed.

### Imaging procedure

Serial dilutions of the prepared drug solutions were transferred into transparent test tubes, and images were taken using a Samsung Galaxy A54 5G smartphone equipped with a 50 MP camera against a white background. All measurements were conducted under constant lighting and fixed photographic conditions, with fixed camera settings. All images were acquired under normal exposure mode with a focal length of 1.57 mm and spot metering to ensure consistent light measurement. The captured images were saved in TIFF format with a resolution of 2604 × 4624 pixels, and color information was represented using the standard RGB color model.

Captured images were processed with ImageJ software, where the average gray intensity values were determined for quantitative evaluation.

### Analysis of pharmaceutical formulation

Ten Alzmenda^®^ tablets, each labeled to contain 10 mg of MEM, were finely powdered. An accurately weighed portion equivalent to one tablet was transferred into a 100 mL volumetric flask, dissolved in distilled water, and the volume was made up to the mark. The solution was sonicated for 5 min and filtered to obtain a clear solution. The resulting stock solution had a concentration of 100 µg mL⁻¹.

Aliquots of 0.5, 1.0, and 2.0 mL of this stock solution were then transferred and subjected to the derivatization procedure described in Sect. “Derivatization procedure for memantine”.

## Results and discussion

**Fig. 1:**
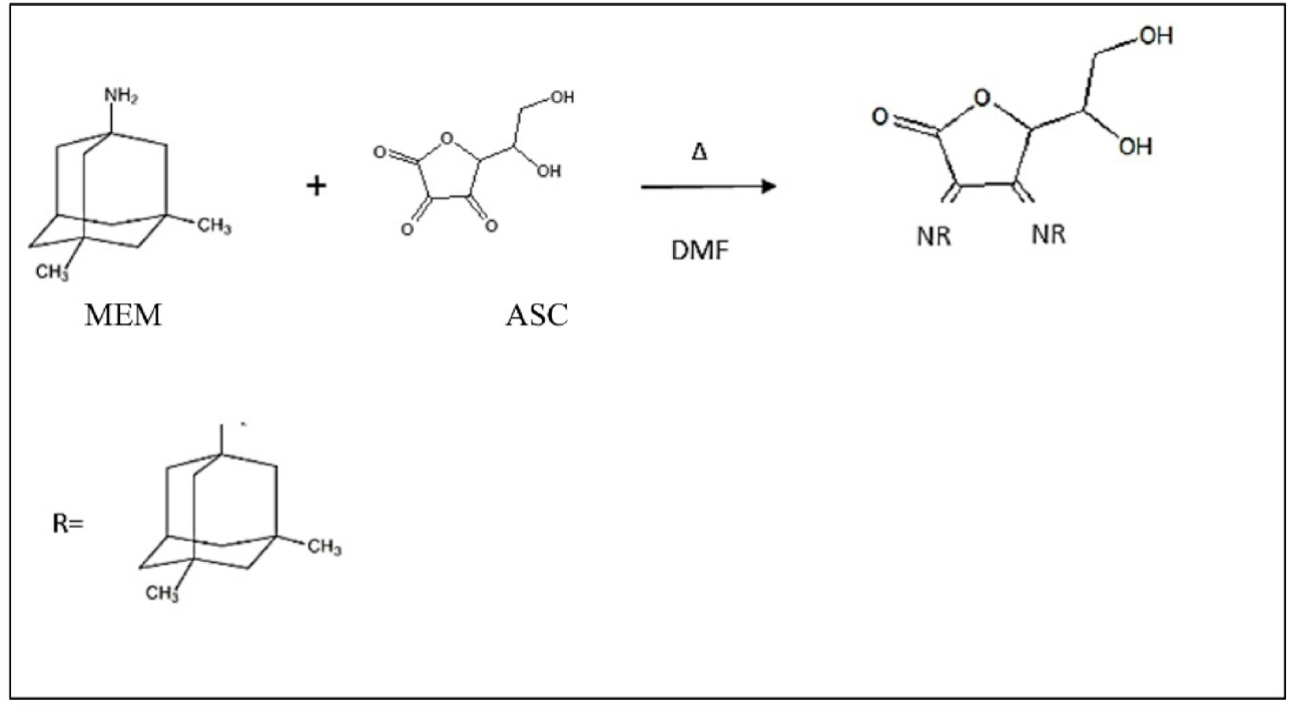
Proposed reaction pathway between MEM and ASC.

**Fig. 2:**
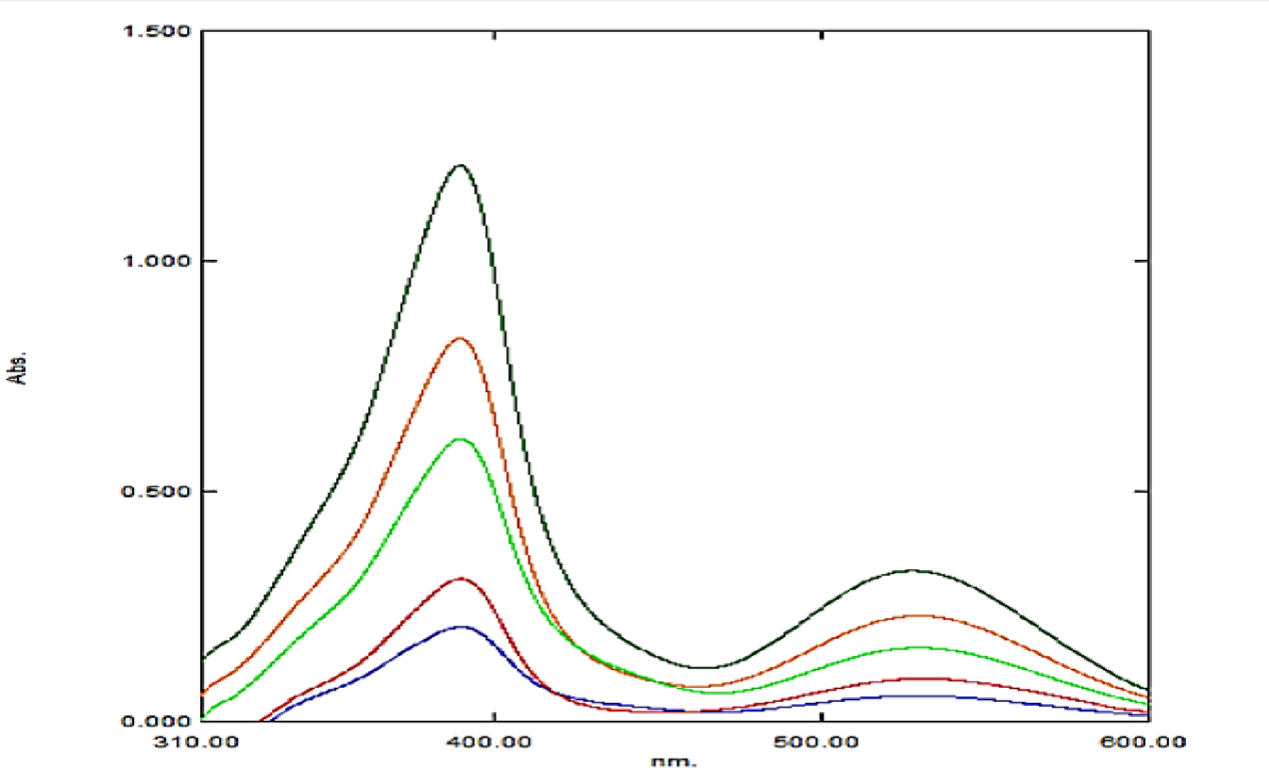
Absorption spectra of MEM–ASC reaction product.

ASC is known to react with aliphatic amines via schiff’s base condensation reaction to form colored products, a reaction that has been successfully utilized for the spectrophotometric determination of various pharmaceuticals. In the present study, MEM reacted with ASC to yield a pink-colored product, as illustrated in Fig. 1. The resulting product exhibited two distinct absorption maxima (λ_max) at 386 nm and 530 nm, as shown in Fig. 2. The wavelength of 386 nm was selected instead of 530 nm for spectrophotometric analysis because it provides a higher absorbance, thereby improving the sensitivity of the assay.

**Fig. 3:**
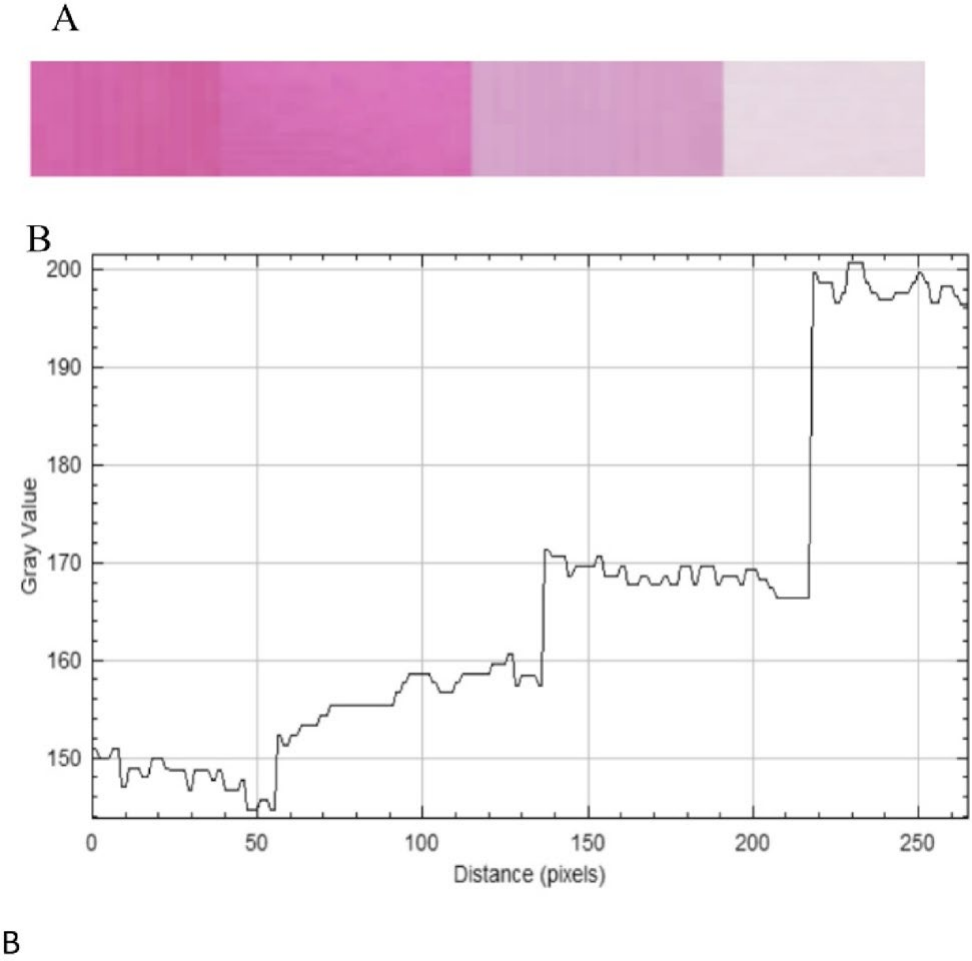
**(a)** Images of successive concentrations of MEM–ASC product (50, 25, 10, and 5 µg mL⁻¹). **(b)** Corresponding gray value plot obtained using ImageJ software.

Based on these findings, a smartphone-assisted colorimetric method was employed using ImageJ software to analyze the captured images of the colored solutions Fig. 3.

The software determined the RGB (red–green–blue) gray values across the image pixels, and the results were plotted as gray value versus pixel position (Fig. 2). The RGB gray value represents the intensity of color for each pixel, where 0 corresponds to black and 255 to white. To better correlate the measured values with the observed color intensity, the RGB data were converted into their complementary CMY (cyan–magenta–yellow) format using the relation CMY = 255 – RGB.

The RGB model was chosen as it provides the highest sensitivity and a high correlation coefficient compared to HSV model, enabling reliable quantitative analysis, because individual color channels can be selectively correlated with analyte concentration, maximizing signal variation while minimizing background interference Table S1. The RGB (Red–Green–Blue) color model is an additive color model used to represent colors through the combination of red, green, and blue light intensities. In smartphone-based colorimetric analysis, each captured image is composed of pixels, and every pixel contains three numerical values corresponding to the intensity of the R, G, and B channels, each ranging from 0 to 255, where 0 represents black and 255 represents the maximum color intensity. The distribution of these RGB values reflects the color characteristics of the analyzed solution. During analysis, the software extracts the RGB gray values from the selected region of interest (ROI) in the captured image. Changes in analyte concentration lead to variations in the color intensity of the solution, which in turn cause measurable changes in the RGB channel values.

### Optimization of reaction conditions

The reaction between MEM and ASC was systematically optimized to achieve maximum color intensity and greater sensitivity.

#### Effect of ascorbic acid volume

Different volumes of ASC solution (0.5–3.0 mL) were tested while keeping other parameters constant. The results indicated that the maximum color development was achieved using 2.0 mL of ASC, which was therefore selected as the optimal reagent volume for subsequent experiments Figure S2.

#### Effect of temperature

The influence of temperature on the reaction was investigated over the range of 40–100 °C. The intensity of the pink-colored MEM–ASC derivative increased with rising temperature, reaching a maximum at 100 °C. Consequently, 100 °C was chosen as the optimal reaction temperature for all further measurements Figure S3.

#### Effect of reaction time

The influence of reaction time on the formation of the pink-colored product was investigated over the range of 10–50 min at the previously optimized reagent volume and temperature. The color intensity increased with time, reaching a maximum at 30 min. Longer reaction times did not lead to a significant increase in absorbance, indicating that 30 min is the optimal reaction time for complete derivatization and maximum analytical sensitivity Figure S4.

### Optimization of imaging procedure

To ensure reliable and reproducible smartphone-assisted colorimetric analysis, the imaging procedure was systematically optimized by evaluating Camera resolution. Three different smartphones with camera resolutions of Samsung Galaxy A12 (13 MP), Samsung Galaxy A32 (25 MP), and Samsung Galaxy A54 (50 MP). were tested. The 50 MP cameras provided more consistent and accurate gray value measurements, whereas the 13, 25 MP cameras showed slightly higher variability. Several distances from the smartphone camera to the test tube were evaluated, and a distance of 10 cm was ultimately chosen for image acquisition. The details of the imaging setup are summarized in Table 1.

**Table 1:** Imaging conditions for MEM–ASC colorimetric analysis.

Parameter	Setting
Camera	50 MP smartphone camera
Focal length	1.57 mm
Exposure mode	Normal
Metering mode	Spot metering
Image format	TIFF
Resolution	2604 × 4624 pixels
Color model	RGB
Imaging distance	10 cm
Background	White in imaging box
Illumination	Standardized lightbox (white LED, fixed intensity)
ROI selection	Averaged region in ImageJ
Size and location	a central square region of 100 × 100 pixels
White balance	Locked to standard daylight setting

### Method validation

The analytical performance of the proposed analytical methods (smartphone based and conventional spectrophotometric approaches) were evaluated following the ICH Q2(R2)^32^ guidelines in terms of linearity, accuracy, precision, and specificity.

#### Linearity

Both the smartphone-assisted colorimetric and traditional spectrophotometric approaches exhibited a linear response over the concentration range of 5.0–50 µg mL⁻¹. For the spectrophotometric approach, calibration was performed by plotting the absorbance at 386 nm versus MEM concentration. For the smartphone-based method, the CMY gray values were plotted against concentration. The corresponding regression parameters are summarized in Table 2.

**Table 2:** Regression parameters for determination of MEM by smartphone assisted and conventional spectrophotometric approaches.

Parameters	Smartphone assisted approach	Spectrophotometric approach
Linearity (µg/mL)	5.0–50	5.0–50
Limit of detection (LOD) (µg/mL)	0.45	0.0615
Limit of quantitation (LOQ) (µg/mL)	1.36	0.19
Slope ± S_b_	2.47 ± 0.011	0.22 ± 0.0013
Intercept ± S_a_	41.22 ± 0.34	0.099 ± 0.0041
S_y/x_	0.43	0.0052
Correlation coefficient (r)	0.9999	0.9999

The limit of detection (LOD) and limit of quantitation (LOQ) were calculated for both methods. The spectrophotometric approach showed LOD and LOQ values of 0.0615 µg mL⁻¹ and 0.19 µg mL⁻¹, respectively, while the smartphone-assisted method yielded LOD and LOQ of 0.45 µg mL⁻¹ and 1.36 µg mL⁻¹, respectively. Indeed, the smartphone-based colorimetric method is inherently less sensitive than UV spectrophotometry, as it relies on the detection of color intensity changes captured by a smartphone camera. At very low concentrations, the pink color formed from the reaction gradually fades and cannot be accurately detected by the smartphone imaging system. Therefore, the UV spectrophotometric method exhibits lower LOD and LOQ values.

#### Accuracy

Accuracy was evaluated by analyzing three different concentrations of MEM (5–20-50 µg mL⁻¹) covering the entire linearity range in triplicate. The methods demonstrated excellent accuracy, as indicated by percent recoveries (98–102%) summarized in Table 3.

**Table 3:** Evaluation of the accuracy results for determination of MEM using smartphone assisted and conventional spectrophotometric approaches.

Conc. taken	Smartphone assisted approach		Spectrophotometric approach	
	Mean* Conc.	Mean* % Recovery	Mean* Conc.	Mean* %Recovery
**5.0**	4.95	99	5.04	100.91
**20**	19.72	99.87	19.97	99.87
**50**	49.09	98.19	50.5	101.01

*Average of three determinations.

#### Precision

Precision was assessed in terms of intra-day (repeatability) and inter-day (intermediate precision). Three concentrations of MEM (10–25-40 µg mL⁻¹) were analyzed in triplicate within the same day for assessment of intra-day precision. The same three concentrations were measured on three days for evaluation of inter-day precision. The results, presented in Table 4, showed low relative standard deviation (RSD) values less than 2%, confirming the high precision of both approaches.

**Table 4:** Evaluation of the precision results for determination of MEM using smartphone assisted and conventional spectrophotometric approaches.

Conc. taken	Smartphone assisted approach				Spectrophotometric approach			
	Intraday		Interday		Intraday		Interday	
	Mean* % Recovery	%RSD	Mean* % Recovery	%RSD	Mean* % Recovery	%RSD	Mean* % Recovery	%RSD
**10**	98.75	1.59	99.61	1.66	99.55	0.87	100.35	1.15
**25**	100.92	1.39	100.73	1.74	100.03	0.71	99.96	1.08
**40**	101.5	0.86	99.43	1.1	100.16	0.56	99.98	1.04

*Average of three determinations.

#### Specificity

**Fig. 4:**
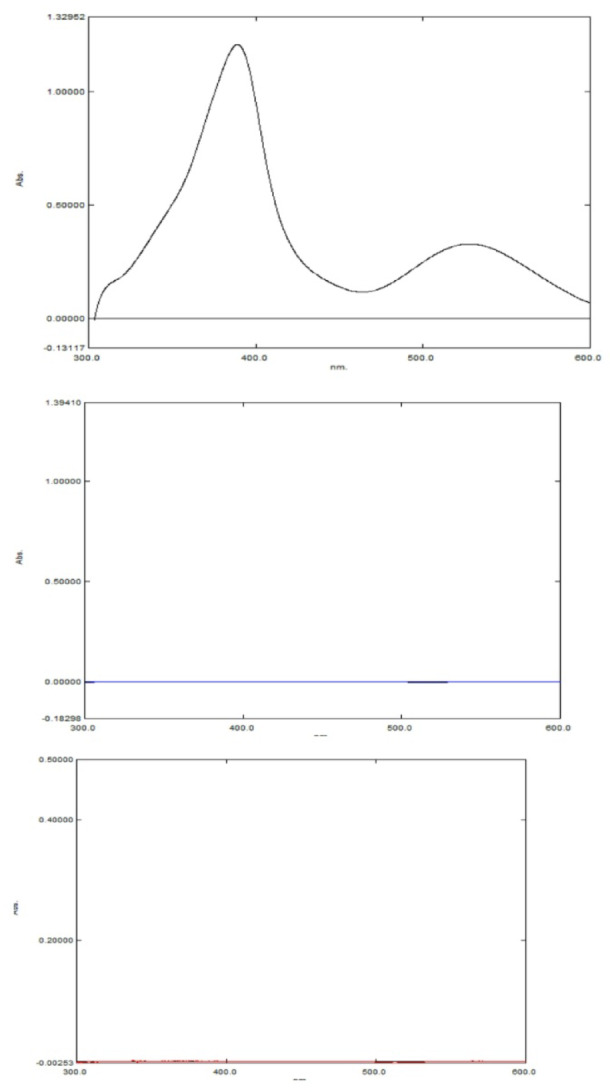
The UV spectra of the MEM–ascorbic acid complex (black), blank ascorbic acid solution (blue), and tablet excipient mixture (red) recorded under the same experimental conditions.

The specificity of the smartphone based and conventional spectrophotometric methods was verified by applying them to commercial Alzmenda^®^ tablets. No interference from tablet excipients was observed, and good percent recoveries. This was further confirmed by Fig. 4, which shows the overlaid UV spectra of the MEM–ascorbic acid complex, blank ascorbic acid solution, and excipient mixture, demonstrating the absence of spectral interference. The accuracy and precision of the results for determination of MEM in dosage form obtained by the two approaches were statistically compared with a previously reported colorimetric method using ninhydrin^3^. The calculated F and t values were less than the corresponding tabulated values, confirming that the newly developed methods are comparable in analytical performance to the reported method as illustrated in Table 5.

**Table 5:** Assay results for the determination of MEM in tablets and statistical comparison to a reported method.

Method	Mean % recovery* ±SD	t-value (2.77) ^a^	F-value (19) ^b^
**Smartphone assisted approach**	99.02 ± 0.84	1.98	1.11
**Spectrophotometric approach**	100.59 ± 0.63	0.28	1.97
**The reported method** ^3^	100.42 ± 0.88		

a, b theoretical values t- value and F- value (0.05) at *n* = 3.

#### Robustness

Robustness of the MEM–ASC colorimetric assay was assessed by introducing small deliberate variations in key experimental parameters. The effect of variations in reagent volume (± 0.1 mL), heating time (± 2 min), heating temperature (± 5 °C), and imaging distance (± 1 cm) on the measured absorbance/color intensity was evaluated in triplicate. Percent relative standard deviation (%RSD) was used to assess robustness Table 6.

**Table 6:** Evaluation of the robustness for determination of MEM using smartphone assisted and conventional spectrophotometric approaches.

Parameter Varied	Variation	%RSD (Smartphone)	%RSD (Spectrophotometry)
ASC reagent volume	1.9 mL	0.98%	0.75%
	2.0 mL	0.91%	0.82%
	2.1 mL	1.1%	0.90%
Heating temperature	95 °C	0.45%	0.95%
	100 °C	0.57%	0.82%
	105 °C	0.38%	0.70%
Heating time	28 min	0.78%	0.54%
	30 min	0.97%	0.62%
	32 min	0.48%	0.43%
Imaging distance	9 cm	0.89%	………
	10 cm	0.57%	………….
	11 cm	0.81%	………….

## Sustainability assessment using the EPPI metric

The sustainability and practicality of the smartphone-based approach and the traditional spectrophotometric method were evaluated using the novel Environmental, Performance, and Practicality Index (EPPI), reported in December 2025^33,34^. EPPI introduces a dual-index system consisting of the Environmental Impact (EI) Index and the Performance and Practicality Index (PPI). The EI Index measures environmental sustainability in accordance with the principles of Green Analytical Chemistry (GAC) and Green Sample Preparation (GSP). It is composed of four key components: sample preparation (S), instrumentation (I), reagents (R), and waste generation (W). The PPI complements the EI Index by assessing analytical performance (redness) and practicality (blueness).

**Fig. 5:**
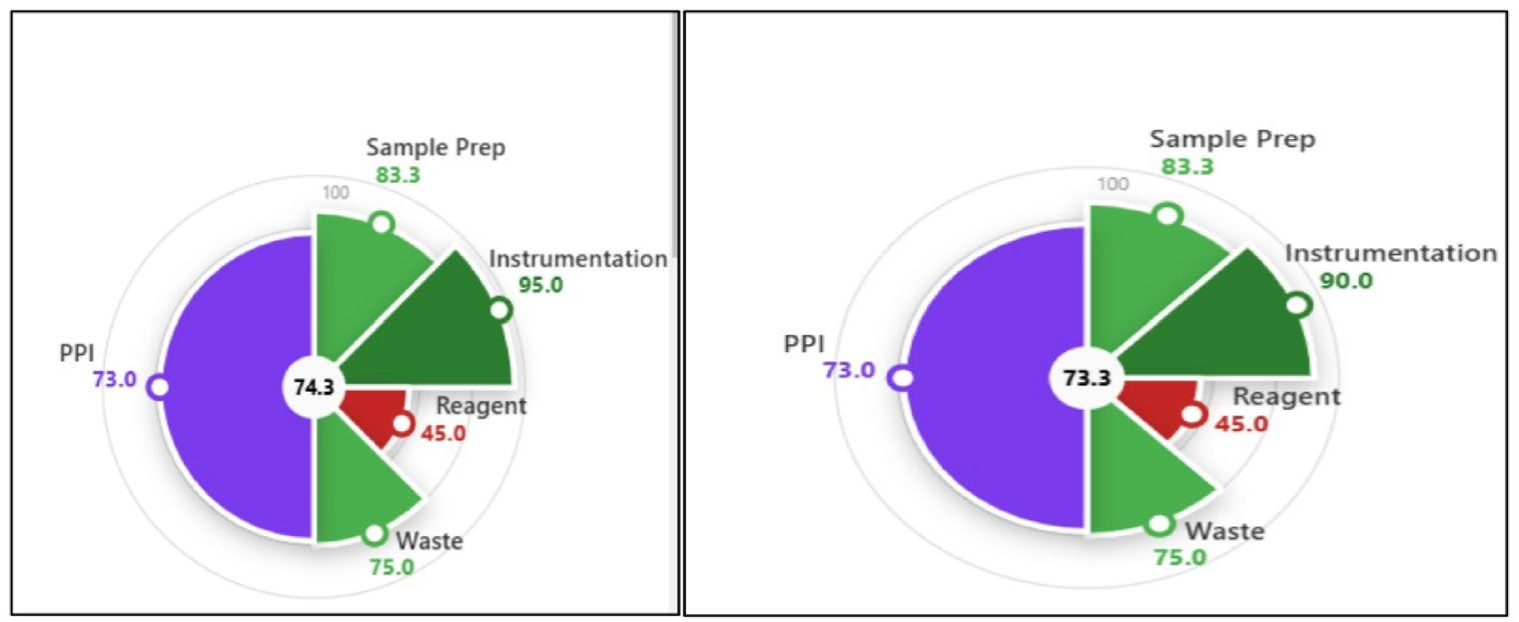
The results for EPPI evaluation of **(a)** the smartphone assisted and **(b)** the spectrophotometric approaches.

EPPI results are presented as both a numerical score (1–100) and a visual pie chart, in which green represents greenness and purple reflects the combined contribution of performance and practicality. Both methods achieved the same practicality score; however, the main difference lies in the instrumentation component of the EI Index. The smartphone-based approach achieved an instrumentation score of 95, while the spectrophotometric method scored 90. This difference resulted in a total EPPI score of 74.6 for the smartphone-based method and 73.3 for the spectrophotometric method, confirming the superior sustainability of the smartphone approach (Fig. 5). A detailed explanation of the EI sub-scores for sample preparation, instrumentation, reagents, and waste is provided in Table 7. The greenness of smartphone approach was also evaluated using established green metric analytical ecoscale^34^ giving a total score of 88, indicating excellent greenness Table 8.

**Table 7:** EI score for smartphone assisted and the spectrophotometric approaches.

Criteria	Smartphone assisted method	spectrophotometric method
**Sample preparation score**	No pre synthesis, minimal sample preparation for UV or smartphone approach, offline, extraction from tablets using non green solvent with volume more than 10mL Total score of 83.3	
**Instrumentation score**	Smartphone approach, Portable, Manual→ **Score: 95**	≤ 0.1 kWh/sample, **Score: 90**
**Reagent score**	Ascorbic acid 0 DMF 1-10mL (3pictograms and danger) → **Score: 45**	
**Waste score**	Waste: 1–10mL, non-biodegradable, no treatment → 90–15 = **75**	
**Total scores/4 and significance**	298.3/4 = 74.6, the method is green	293.3/4 = 73.3 the method is green

**Table 8:** Calculations for analytical ecoscale score for the developed smartphone and spectrophotometric approach.

Parameters	Penalty Points (PPs)	
	Smartphone assisted method	Spectrophotometric method
Reagents		
Type (Hazard)	DMF (**3 Pictograms**,** Danger**) 1–10 ml (2*3*1 = 6)	
Amount (hazard* amount)		
Instrument		
Energy consumption	Smartphone (**0**)	< 0.1 kW/h (**0)**
Emission of vapors or gasses	0	0
Waste		
Waste generated	1 to 10 mL (**3**) No treatment (**3**)	
Waste treatment		
Total PPs	**12**	
Score (100-PPs)	**88**	

## Conclusion

In conclusion, the smartphone-based colorimetric method has comparable analytical performance to the traditional spectrophotometric approach and demonstrates strong potential for the application of pharmaceutical sensing in different matrices as an eco-friendly and cost-effective tool. Herein, we developed simple and reliable methods for the determination of MEM using either smartphone-assisted colorimetric analysis or traditional spectrophotometry. ASC was utilized as a derivatizing reagent, which reacts with MEM to produce a pink-colored product. Both approaches demonstrated good linearity, accuracy, precision, and specificity, making them suitable for routine analysis of MEM in pharmaceutical formulations. The method was carefully optimized with respect to reagent volume, temperature, and reaction time, ensuring maximum color development and analytical sensitivity. Evaluation using the EPPI tool, which considers greenness, applicability, and analytical performance, showed higher total scores for the smartphone-based method, highlighting its superior sustainability and practicality.

## Electronic Supplementary Material

Below is the link to the electronic supplementary material.

Supplementary Material 1

## Data Availability

All data generated or analysed during this study are included in this published article [and its supplementary information files].
