# Supplementary material for: Smartphone based colorimetric method for determination of memantine via schiff’s base reaction with ascorbic acid
Source: Sci Rep. 2026 May 12;16:14739. doi: 10.1038/s41598-026-51216-4 (PMC13168557; doi:10.1038/s41598-026-51216-4)
Supplement: Supplementary file 1 — Supplementary Material 1 [file 41598_2026_51216_MOESM1_ESM.docx]

**Smartphone Based Colorimetric Method for Determination of Memantine via Schiff’s Base Reaction with Ascorbic Acid**

**Supplementary information**


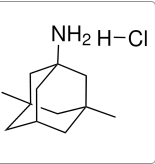


**Figure S1: Chemical structure of Memantine hydrochloride**

**
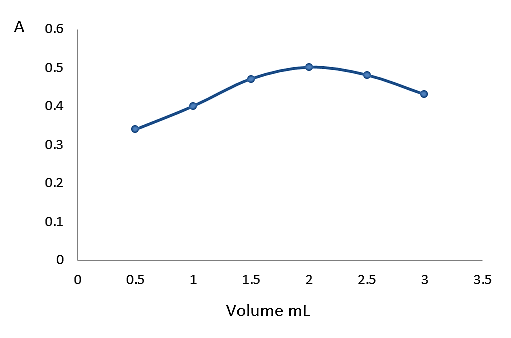
**

**Figure S2: The effect of the volume of ASC on the reaction with MEM 25μ/mL**

**
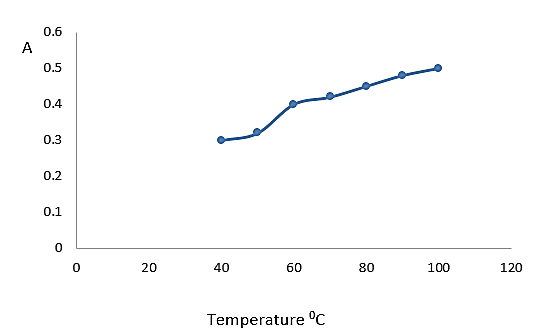
**

**Figure S3: The effect of the temperature on the reaction of MEM 25μ/mL with ASC**

**
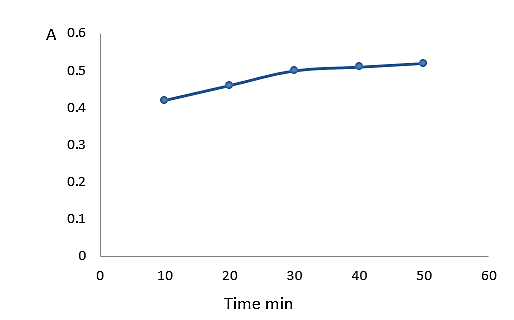
**

**Figure S4: The effect of the heating time on the reaction of MEM 25μ/mL with ASC**

**Table S1: RGB model Data**

| **Concentration (µg/mL)** | **R** | **G** | **B** | **Mean RGB** |
| --- | --- | --- | --- | --- |
| 5 | 105 | 115 | 120 | 113.3 |
| 10 | 120 | 130 | 135 | 128.3 |
| 20 | 140 | 150 | 155 | 148.3 |
| 30 | 160 | 170 | 175 | 168.3 |
| 40 | 180 | 190 | 195 | 188.3 |
| 50 | 200 | 210 | 215 | 208.3 |
